# DKB114, A Mixture of *Chrysanthemum Indicum* Linne Flower and *Cinnamomum Cassia* (L.) J. Presl Bark Extracts, Improves Hyperuricemia through Inhibition of Xanthine Oxidase Activity and Increasing Urine Excretion

**DOI:** 10.3390/nu10101381

**Published:** 2018-09-28

**Authors:** Young-Sil Lee, Seung-Hyung Kim, Heung Joo Yuk, Dong-Seon Kim

**Affiliations:** 1 Herbal Medicine Research Division, Korea Institute of Oriental Medicine, 1672 Yuseong-daero, Yuseong-gu, Dajeon 34054, Korea; rheeys04@kiom.re.kr (Y.-S.L.); yukhj@kiom.re.kr (H.J.Y.); 2 Institute of Traditional Medicine and Bioscience, Daejeon University, 62 Daehak-ro, Dong-gu, Daejeon 34520, Korea; sksh518@dju.kr

**Keywords:** *Chrysanthemum indicum* Linne flower, *Cinnamomum cassia* (L.) J. Presl bark, hyperuricemia, xanthine oxidase, urate transporters

## Abstract

*Chrysanthemum indicum* Linne flower (CF) and *Cinnamomum cassia* (L.) J. Presl bark (CB) extracts have been used as the main ingredients in several prescriptions to treat the hyperuricemia and gout in traditional medicine. In the present study, we investigated the antihyperuricemic effects of DKB114, a CF, and CB mixture, and the underlying mechanisms in vitro and in vivo. DKB114 markedly reduced serum uric acid levels in normal rats and rats with PO-induced hyperuricemia, while increasing renal uric acid excretion. Furthermore, it inhibited the activity of xanthine oxidase (XOD) in vitro and in the liver in addition to reducing hepatic uric acid production. DKB114 decreased cellular uric acid uptake in oocytes and HEK293 cells expressing human urate transporter (hURAT)1 and decreased the protein expression levels of urate transporters, URAT1, and glucose transporter, GLUT9, associated with the reabsorption of uric acid in the kidney. DKB114 exerts antihyperuricemic effects and uricosuric effects, which are accompanied, partially, by a reduction in the production of uric acid and promotion of uric acid excretion via the inhibition of XOD activity and reabsorption of uric acid. Therefore, it may have potential as a treatment for hyperuricemia and gout.

## 1. Introduction

Hyperuricemia is characterized by elevated blood uric acid levels [1], which cause accumulation of urate crystals in joints and the kidney, leading to gout and gouty arthritis [2,3]. The prevalence of hyperuricemia and gout is increasing worldwide, and uric acid is a risk factor of fatty liver, insulin resistance, hypertension, and cardiovascular diseases [4]. Therefore, there is a growing interest in hyperuricemia and uric acid regulation. Hyperuricemia is caused by increased production or impaired uric acid excretion or a combination of these two mechanisms [5,6]. Uric acid is produced by the activities of xanthine oxidase (XOD), a key enzyme that converts hypoxanthine and xanthine, which is then converted to uric acid in purine metabolism [7]. The elimination of uric acid occurs via complex urate transporters that regulate its reabsorption and secretion, such as apical urate/anion exchanger, URAT1, and basolateral glucose transporter 9 (GLUT9) in the kidney [8]. Accordingly, reducing uric acid production and increasing uric acid excretion may be useful therapeutic approach for hyperuricemia treatment. Currently, XOD inhibitors such as allopurinol (AP) and febuxostat, and uricosuric agents such as benzbromarone (Ben) and probenecid, are used as antihyperuricemia drugs for clinical hyperuricemia treatment [9]. However, these drugs are poorly tolerated and induce side effects, such as drug allergies, gastrointestinal symptoms, kidney diseases, hypersensitivity syndrome, and hepatotoxicity. These adverse effects limit the use of these drugs [10,11,12,13]. Thus, new therapeutic strategies with minimal side effects are needed. An increasing interest in natural products, such as herbal medicines, has elucidated new approaches that can overcome these limitations [13], making natural products a promising pool of candidates for drugs and functional foods for hyperuricemia management.

*C. indicum* and its flowers have been used to treat inflammation-related diseases, infectious diseases, hypertension, eye diseases, and headaches in Korea and China. It has various biological properties such as antioxidation and antitumor properties [14,15]. *C. cassia* bark has been used to treat immune-related diseases, gastritis, diarrhea, and cancer in traditional medicine and has been shown to have anticancer and antidiabetic effects [16,17]. In addition, *C. indicum* flower and *C. cassia* bark inhibited XOD activity in vitro, and decreased serum uric acid levels in animal models of potassium oxonate (PO)-induced hyperuricemia [14,18,19,20]. Interestingly, in our previous study, we demonstrated the antihyperuricemic effect of *C. indicum* flower and *C. cassia* bark as well as the synergistic antihyperuricemic effect of their mixture [21]. Furthermore, we found that the mixture and its major component inhibited XOD activity in vitro. However, there was no evidence of uric acid excretion induced by the mixture. Therefore, in the present study, we performed a series of experiments to further the understanding of the mechanism underlying the antihyperuricemic effects of *C. indicum* flower and *C. cassia* bark mixture, DKB114, with a focus on XOD activity and uric acid excretion.

## 2. Materials and Methods

### 2.1. DKB114 Preparation

*C. indicum* Linne flowers (CF) were purchased from Uiseong in Korea. *C. cassia* (L.) J. Persl barks (CB) were purchased from Yen Bai of Veitnam. CF and CB were identified by the Classification and Identification Committee of the Korea Institute of Oriental Medicine (KIOM) and their voucher specimens (No. GHP-030 and 78) were stored at the herbarium of the Department of Herbal Resources Research of KIOM. Ratio of *C. indicum* flower and C. cassia bark mixture, DKB114 was selected and prepared by mixing previously prepared *C. indicum* flower and C. cassia bark extracts at a weight ratio of 1:2 based on our previous report [22]. 

### 2.2. Animals

Sprague-Dawley rats (male, age: 7 weeks) were purchased from Orient Bio (Seongnam, Korea) and maintained in a room under the following conditions: A temperature of 22 ± 1 °C, humidity of 50 ± 10%, and a 12-h light/dark cycle. The rats were allowed free access to diet (AIN-76A, Research Diet, New Brunswick, NJ, USA) and water. The experimental method was approved by the Committee on Animal Care of Korea Institute of Oriental Medicine, and all experiments were performed in accordance with the committee guidelines (Approval No.17-067)

### 2.3. Sample Treatment in Normal Rats

The rats were divided into the following five groups (*n* = 8/group): (1) Normal control (NC) group treated with the vehicle (0.5% CMC solution), (2) 100 mg/kg DKB114 group, (3) 150 mg/kg DKB114 group, (4) 200 mg/kg DKB114 group, and (5) 10 mg/kg AP group. The DKB114 and AP groups were administered with DKB114 and AP at the aforementioned doses in 0.5% CMC solution.

### 2.4. Hyperuricemia Induction and Sample Treatment

To induce hyperuricemia, the uricase inhibitor PO was injected, as previously described [22], with slight modifications. For the experiment on the dose-dependent effects of DKB114 (Exp1), the rats were randomized into the following six groups (*n* = 10/group): (1) NC, (2) hyperuricemia control group (PO), (3) PO + 100 mg/kg DKB114 group, (4) PO + 150 mg/kg DKB114 group, (5) PO + 200 mg/kg DKB114 group, and (6) PO + 10 mg/kg AP group. Next, for the experiment on the uric acid excretion-promoting effect of DKB114 (Exp2), the rats were randomized into the following four groups (*n* = 6/group): (1) NC group, (2) PO group, (3) PO + 200 mg/kg DKB114 group, and (4) PO + 50 mg/kg Ben group. In Exp1 and 2, all groups except the NC group were injected intraperitoneally with 150 mg/kg PO, the uricase inhibitor, prepared in 0.5% CMC with 0.1 M sodium acetate (pH 5.0) to induce hyperuricemia. The NC group received 0.5% CMC with 0.1 M sodium acetate. In Exp1, groups (1) and (2) received the vehicle (0.5% CMC) by oral gavage. All test samples were administered simultaneously along with PO injection. In Exp2, groups (1) and (2) received the vehicle, and groups (3) and (4) were orally treated with 200 mg/kg DKB114 and 50 mg/kg Ben, respectively, for 2 days. PO injection and sample treatments were performed simultaneously for all animals. 

### 2.5. Collection of Serum, Urine, and Tissues 

Urine was collected using metabolic cages at 1, 2, 3, and 4.5 h after PO injection and sample administration on the first day. Collected urine samples were and centrifuged (3000× *g*, 10 min, 4 °C) to remove particulate contaminants. The supernatants were stored at −80 °C until analysis. Blood was collected via cardiac puncture under anesthesia 2 h after PO injection and DKB114 treatment on the second day. Serum was obtained via centrifugation (3000× *g*, 10 min, 4 °C), and the separated serum was stored at −80 °C until analysis. After blood collection, liver, and kindey tissues were dissected immediately, rinsed, weighed, frozen in liquid nitrogen, and stored at −80 °C until analysis.

### 2.6. Analysis of Uric Acid Levels in Serum, Urine, and Liver Tissues

To analyze hepatic uric acid levels, liver tissues were homogenized in 80 mM potassium phosphate buffer (pH 7.4) and the homogenate was centrifuged (10,000× *g*, 10 min, 4 °C). The resulting supernatant was used to determine the uric acid concentration. Serum, urine, and liver uric acid levels were measured by using commercial assay kits (Biovision, Milpitas, CA, USA) according to manufacturer’s protocols.

### 2.7. In Vitro and In Vivo XOD Inhibition Assay

For the analysis of the in vitro XOD activity, the reaction mixture comprising 50 mM sodium phosphate buffer (pH 7.6), 17.9 nM xanthine sodium salt, 0.04 units of xanthine oxidase, and DKB114 at various concentrations was analyzed for decreased uric acid production at 295 nm. AP served as a positive control for XOD activity analysis. All experiments were performed in triplicate. Liver XOD activity was measured using commercial assay kits (Sigma, Saint Louis, MO, USA) according to the manufacturer’s protocols. Protein concentrations were measured by bicinchoninic acid protein assays using bovine serum albumin (BSA) as the standard to normalize XOD activity. XOD activity was presented as nanomoles of uric acid formed per minute per milligram of protein.

### 2.8. Western Blotting Analysis of Kidney Samples

Proteins from kidney tissues were extracted using RIPA buffer (ATTO Corporation, Tokyo, Japan), and protein concentrations were determined by BCA protein assays (Thermo Fisher Scientific, Waltham, MA, USA) using BSA as a standard. Proteins were separated by 10% SDS polyacrylamide gel electrophoresis and were electroblotted onto a nitrocellulose membrane (Amersham, Piscataway, NJ, USA). The membranes were blocked with EzBlockChemi blocking solution (ATTO Corporation, Tokyo, Japan) and subsequently incubated with anti-URAT1 (MyBioSource.com, San Diego, CA, USA); organic anion transporter (OAT)1, GLUT9, OAT3, and GAPDH primary antibodies and a horseradish peroxidase-conjugated secondary antibody (Santa Cruz Biotechnology, Santa Cruz, CA, USA). The immunocomplexes were developed by enhanced chemiluminescence (Amersham, Buckinghamshire, UK) and exposed to ImageQuant LAS 4000 system software (GE Healthcare, Chicago, IL, USA). The images obtained were subjected to a densitometric analysis using Image J1.49 software (http://rsb.info.nih.gov/ij/download.html; National Institute of Health, National Institute of Health, Bethesda, MD, USA). The URAT1, GLUT9, OAT1, and OAT3 protein expression levels were presented relative to those in the PO group after normalization to the GAPDH protein levels.

### 2.9. Cell Culture

Human embryonic kidney (HEK) 293 cells and HepG2 cells were cultured in Dulbecco’s modified Eagle’s medium (DMEM, Gibco BRL, Waltham, MA, USA) supplemented with 10% fetal bovine serum (Gibco BRL) and antibiotics (Gibco BRL) at 37 °C in a humidified 5% CO_2_ incubator.

### 2.10. Establishment of hURAT1-Expressing Oocytes and HEK293 Cells

To establish hURAT1-expressing oocytes, a pcDNA3.1 (+) expression vector containing the cDNA of hURAT1 (GenBank accession number AB071863) was linearized using restriction enzymes NheI and XbaI and then transcribed into complementary RNA (cRNA). cRNA was microinjected into Xenopus laevis oocytes. The hURAT1-expressing oocytes were used for a uric acid uptake experiment at 2 days after microinjection. To establish hURAT1-expressing HEK293 cells, the cDNA of hURAT1 (GenBank accession number AB071863) from the human kidney was subcloned into pcDNA 3.1 (+) (Invitrogen, Carlsbad, CA, USA) using restriction enzymes NheI and XbaI. HEK293-URAT1 cells were obtained by transient transfection of HEK293 cells with a hURAT1 expression vector by using Lipofectamine 2000 according to the manufacturer’s protocols (Invitrogen, Carlsbad, CA, USA). An empty pcDNA 3.1 (+) vector was transfected into the HEK293 cells as a control. The hURAT1-expressing HEK293 cells were used for a uric acid uptake experiment at 1 day after transfection.

### 2.11. Uptake Experiment in hURAT1-Expressing Oocytes and HEK293 Cells

The hURAT1-expressing HEK293 cells and oocytes were pre-incubated in assay buffer with Dulbecco’s phosphate-buffered saline (DPBS, Sigma-Aldrich, Saint Louis, MO, USA) solution and ND96 solution (96 mM NaCl, 2 mM KCl, 1.8 mM CaCl_2_, 1 mM MgCl_2_, and 5 mM HEPES; pH 7.4) supplemented with 1 mM pyrazine carboxylic acid (Sigma-Aldrich, Saint Louis, MO, USA) at 37 °C, respectively. After pre-incubation, the cells were incubated in a solution containing 50 μM (^14^C) uric acid (Moravek Biochemicals, Brea, CA, USA) with various DKB114 concentrations at 37 °C for an additional 10 min for hURAT1-expressing HEK293 cells and 60 min for hURAT1-expressing oocytes. The uric acid uptake was stopped by adding ice-cold DPBS and washing three times with the same solution. The cells were lysed using 0.1 N NaOH with 10% SDS solution, and then radioactivity was determined by liquid scintillation counting. The URAT1 inhibitor Ben (TCI, Tokyo, Japan) was used as the positive control. To determine the cell viability, HEK293 cells were treated with DKB114 at concentration of 10, 50, 100, 200, and 500 μg/mL for 10 min. Cell viability was determined using 3-(4,5-dimethyl-2-thiazolyl)-2,5-diphenyl-2*H*-tetrazolium bromide (MTT) labeling reagent (Cell proliferation kit I, Roche, Basel, Switzerland). 

### 2.12. Mitochondrial Toxicity Study in HepG2 Cells

HepG2 cells were treated with various concentrations of DKB114 (25, 50, 100, 200, and 400 μg/mL) for 4 h. After incubation, mitochondrial membrane potential (MMP) was analyzed using the commercial TMRE-Mitochondrial Membrane Potential Assay Kit (Abcam, Cambridge, MA, USA) and Cell counting kit-8 (Dojindo Molecular Technologies, Inc, Germantown, MD, USA), respectively, according to the manufacturer’s protocol.

### 2.13. Statistical Analysis

Data are expressed as the mean ± SEM. Differences among the treatment groups were analyzed by one-way ANOVA, and a Dunnett’s multiple comparison test was applied to identify the significance using Prism 7.0 software (GraphPad Software Inc., San Diego, CA, USA); and *p* < 0.05 was considered statistically significant.

## 3. Results

### 3.1. Effects of DKB114 on Serum and Urinary Uric Acid Levels in Normal Rats and Rats with PO-Induced Hyperuricemia

**Figure 1:**
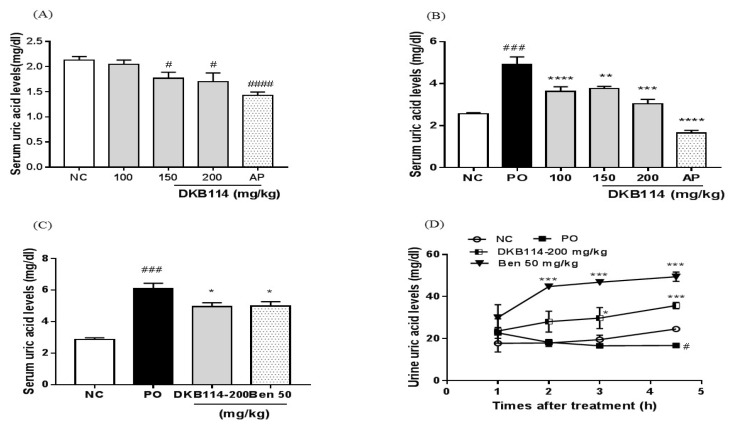
Effects of DKB114 on serum and urinary uric acid levels in normal rats and rats with PO-induced hyperuricemia. (**A**) Serum uric acid levels in normal rats. (**B**) and (**C**) Serum uric acid levels in rats with PO-induced hyperuricemia (Exp1 and 2), and (**D**) urinary uric acid levels in rats with PO-induced hyperuricemia (Exp2). Blood samples were collected 2h after PO injection and DKB114 treatment. Urine samples were collected using metabolic cages at indicated times after PO injection and DKB114 treatment. NC, normal control group; PO, potassium oxonate-induced hyperuricemia group; DKB114, a mixture of ethanol extracts of *Chrysanthemum indicum* Linne flower (CF) and *Cinnamomum cassia* (L.) J. Presl bark (CB) in a 1:2 ratio; AP 10, 10 mg/kg allopurinol; Ben 50: 50 mg/kg benzbromarone. Data are expressed as the mean ± SEM (*n* = 6–10). ^#^ *p* < 0.05, ^###^ *p* < 0.005, and ^####^ *p* < 0.001 vs. the NC group; * *p* < 0.05, ** *p* < 0.01, *** *p* < 0.005, and **** *p* < 0.001 vs. the PO group.

As shown in Figure 1A, 150 and 200 mg/kg DKB114 significantly decreased serum uric acid levels by 16.8% and 20.4%, respectively (both, *p* < 0.05) and 10 mg/kg AP also decreased serum uric acid levels by 32.9% (*p* < 0.005) compared to those in the NC group. Serum uric acid levels were significantly higher in the PO group than in the NC group (*p* < 0.005). On the other hand, DKB114 at doses of 100, 150, and 200 mg/kg significantly reduced serum uric acid levels by 26.2%, 23.5%, and 38.3%, respectively (*p* < 0.005, *p* < 0.01, and *p* < 0.005, respectively), and AP decreased serum uric acid levels by 66.6% compared to those in the PO group (Figure 1B). Furthermore, 200 mg/kg DKB114 markedly reduced serum uric acid levels by 18% (*p* < 0.05, *p* < 0.05, and *p* < 0.01, respectively) and 50 mg/kg Ben, the positive control, decreased serum uric acid levels by 18% compared to those in the PO group in the experiment for uric acid excretion-promoting effect (Figure 1C). At 4.5 h after PO injection, urinary uric acid levels decreased in the PO group compared to those in the NC group (*p* < 0.001). In contrast, 200 mg/kg DKB114 significantly increased urinary uric acid levels by 1.8-fold (*p* < 0.05) and 2.1-fold (*p* < 0.005) at 3 and 4.5 h, respectively, after PO injection. Ben (50 mg/kg) also markedly increased urinary uric acid levels by 2.4-fold (*p* < 0.005), 2.8-fold (*p* < 0.005), and 2.9-fold (*p* < 0.005), respectively, at 2, 3, and 4.5 h after PO injection (Figure 1D).

### 3.2. Effects of DKB114 on XOD Inhibition Activity in Vitro/Vivo and Liver Uric Acid Levels

**Figure 2:**
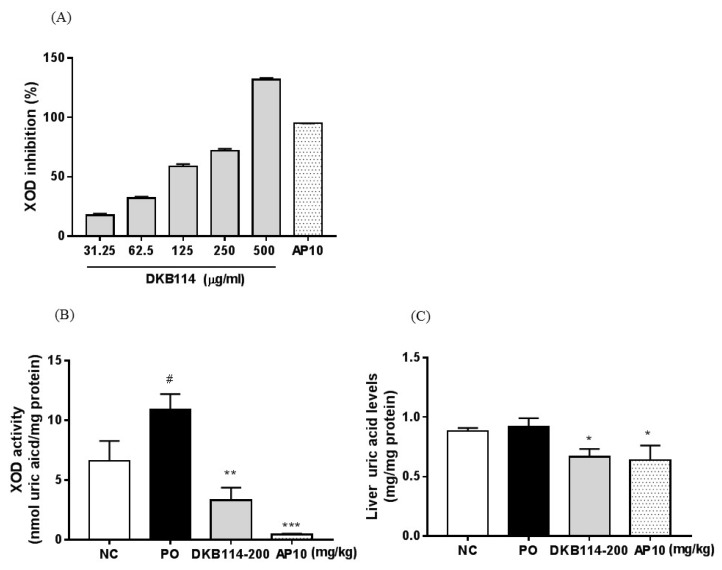
Effects of DKB114 on XOD activity in vitro/in vivo and hepatic uric acid levels. (**A**) In vitro xanthine oxidase (XOD) inhibitory activity, (**B**) liver XOD activity, and (**C**) liver uric acid levels. Liver tissue for analysis of XOD activity and uric acid levels was harvested in Exp1. NC, normal control group; PO, potassium oxonate-induced hyperuricemia group; DKB114, a mixture of ethanol extracts of *Chrysanthemum indicum* Linne flower (CF) and *Cinnamomum cassia* (L.) J. Presl bark (CB) in a 1:2 ratio; AP 10: 10 mg/kg allopurinol. Data are expressed as the mean± SEM (*n* = 4) and representative of three independent experiments in vitro XOD activity and in vivo data are expressed as the mean ± SEM (*n* = 6). ^#^ *p* < 0.05 vs. the NC group; * *p* < 0.05, ** *p* < 0.01, and *** *p* < 0.005 vs. the PO group.

The effects of the DKB114 on in vitro and in vivo XOD inhibition activity are shown in Figure 2. In the in vitro XOD inhibition assay, 31.25, 62.5, 125, 250, and 500 μg/mL DKB114 inhibited XOD activity by 17.6%, 32.1%, 58.8%, 72.0%, and 131.8%, respectively, and the IC_50_ values for DKB114 were 104.4 μg/mL (Figure 2A). The in vivo liver XOD activity in the PO group increased compared to that in the NC group (*p* < 0.05). However, the 200 mg/kg DKB114 and 10 mg/kg AP groups showed markedly reduced XOD activity and inhibited it by 66.7% and 95.6%, respectively (*p* < 0.01 and *p* < 0.005, respectively, compared to that in the PO group (Figure 2B). On the other hand, 200 mg/kg DKB114 was shown to remarkably lower liver uric acid levels compared to those in the PO group (*p* < 0.05). AP (10 mg/kg) also significantly decreased liver uric acid levels (*p* < 0.05) (Figure 2C).

### 3.3. Effects of DKB114 on Uric Acid Uptake in hURAT1-Expressing Oocytes and HEK293 Cells

**Figure 3:**
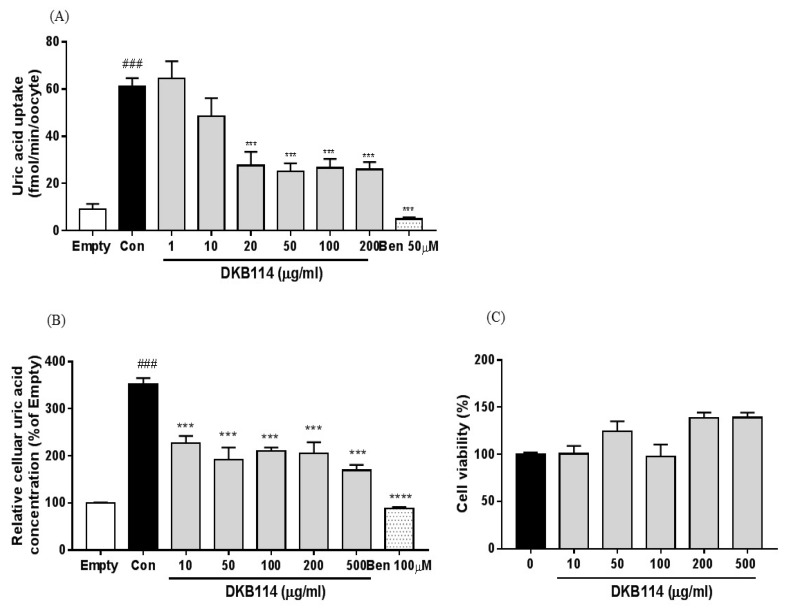
Effects of DKB114 on uric acid uptake in hURAT1-expressing oocytes and HEK293 cells. (**A**) Uric acid uptake in hURAT1-expressing oocytes. (**B**) Uric acid uptake in hURAT1-expressing HEK293 cells. (**C**) Cell viability. Uric acid uptake in hURAT1-expressing oocytes and HEK293 cells expressing was analyzed as described in the Materials and Methods. Empty vector: pcDNA 3.1 vector-transfected oocytes and HEK293 cells; Con: hURAT1-transfected oocytes and HEK293 cells; DKB114, a mixture of ethanol extracts of *Chrysanthemum indicum* Linne flower (CF) and *Cinnamomum cassia* (L.) J. Presl bark (CB) in a 1:2 ratio; Ben: benzbromarone. Data are expressed as the mean ± SEM (*n* = 5) and representative of three independent experiments. ^###^ *p* < 0.005 vs. the Mock; *** *p* < 0.005 vs. the Con.

Uric acid uptake significantly increased by 6.7-fold in the controls (hURAT1-expressing oocytes) compared to that empty vector-transfected oocytes. In contrast, 20, 50, 100, and 200 μg/mL DKB114 treatment significantly reduced cellular uric acid uptake by 55.0%, 59.1%, 56.6%, and 57.6%, respectively (all, *p* < 0.005), compared to that in the controls. Ben (50 μM) also decreased the uric acid uptake (*p* < 0.005) (Figure 3A). In addition, uric acid uptake in control cells (hURAT1-expressing HEK293 cells) significantly increased by 3.5-fold compared to that in empty vector-transfected HEK293cells, whereas 10, 50, 100, 200, and 500 μg/mL DKB114 treatment markedly reduced the uric acid uptake in hURAT1-expressing HEK293 cells by 35.5%, 45.5%, 40.2%, 41.8%, and 51.9%, respectively (all, *p* < 0.005) (Figure 3B). Ben treatment also decreased the uric acid uptake in hURAT1-expressing oocytes and HEK293 cells by 91.8% and 75.0%, respectively (both, *p* < 0.005). As shown in Figure 3C, DKB114 treatment did not show cytotoxicity in HEK293 cells.

### 3.4. Effects of DKB114 on the Expression of Uric Acid Transporters in Rats with PO-Induced Hyperuricemia

**Figure 4:**
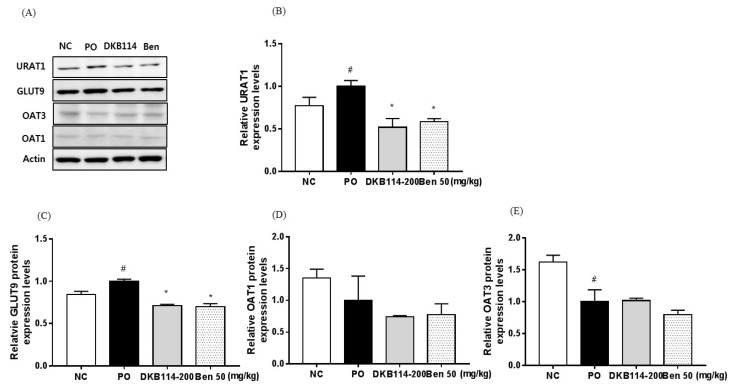
Effects of DKB114 on the expression of uric acid transporters in kidney of rats with PO-induced hyperuricemia. (**A**) Representative proteins expression levels determined by western blot. (**B**) URAT1, (**C**) GLUT9, (**D**) OAT1, and (**E**) OAT3 protein expression levels. Kidney tissue for analysis of protein expression levels was harvested in Exp2. NC, normal control group; PO, potassium oxonate-induced hyperuricemia group; DKB114, a mixture of ethanol extracts of *Chrysanthemum indicum* Linne flower (CF) and *Cinnamomum cassia* (L.) J. Presl bark (CB) in a 1:2 ratio; Ben 50: 50 mg/kg benzbromarone. Data are expressed as the mean ± SEM (*n* = 6). ^#^ *p* < 0.05 vs. the NC group; * *p* < 0.05 vs. the PO group.

URAT1 and GLUT9 protein expression levels were higher in the PO group than in the NC group (*p* < 0.05), but the expression levels were significantly suppressed in the 200 mg/kg DKB114 group and the 50 mg/kg Ben group compared to that in the PO group (all, *p* < 0.05) (Figure 4A–C). OAT1 protein expression levels did not alter among the groups (Figure 3A,D). OAT3 protein expression levels were lower in the PO group than in the NC group (*p* < 0.05), but did not alter among the PO, DKB114, and Ben groups (Figure 4A,E).

### 3.5. Effects of DKB114 on Mitochondrial Toxicity in HepG2 Cells

**Figure 5:**
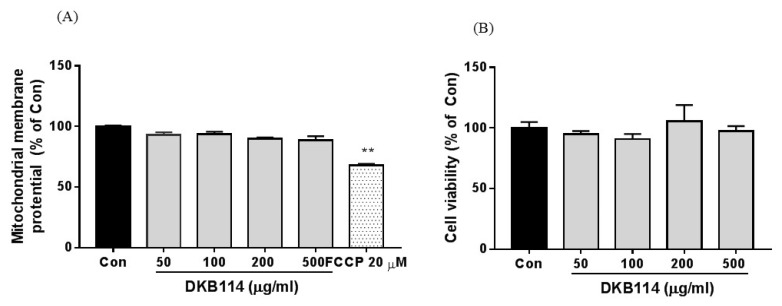
Effects of DKB114 on mitochondrial toxicity in HepG2 cells. (**A**) Mitochondrial membrane potential (MMP) and (**B**) cell viability. MMP and cell viability in HepG2 cells were analyzed as described in the Materials and Methods. Con: untreated cells; DKB114, a mixture of ethanol extracts of *Chrysanthemum indicum* Linne flower (CF) and *Cinnamomum cassia* (L.) J. Presl bark (CB) in a 1:2 ratio. FCCP: Carbonyl cyanide 4-(trifluoromethoxy) phenylhydrazone). Data are expressed as the mean ± SEM (*n* = 4) and are representative of three independent experiments. ). ** *p* < 0.01 vs. the control group.

It is known that Ben has hepatic toxicity, and mitochondrial toxicity has been proposed as the underlying mechanism of hepatic toxicity. To assess the mitochondrial toxicity of DKB114 treatment, we determined the MMP in HepG2 cells. As shown in Figure 5A, DKB114 treatment did not change the MMP. Additionally, DKB114 did not affect cell viability (Figure 5B).

## 4. Discussion

In our previous study, we found that DKB114, a combination of CF and CB extracts, exerted antihyperuricemic effects in rat models of PO-induced hyperuricemia. Based on our previous research, we further study the understanding of the mechanism underlying the antihyperuricemic effects. In this study, we found that DKB114 significantly decreased serum uric acid levels in normal rats and rats with PO-induced hyperuricemia, consistent with that of our previous study and other studies [18,19,20,21]. Furthermore, DKB114 increased uric acid levels in urine, indicating that DKB114 has the ability to increase uric acid excretion and that it might be a potent uricosuric agent, which may explain its antihyperuricemic effects.

Generally, hyperuricemia in humans can be divided into two categories based on pathophysiology, uric acid overproduction, and underexcretion. Uric acid production is mainly regulated by XOD, a key enzyme that converts hypoxanthine and xanthine to uric acid [23,24]. Thus, inhibition of XOD activity should be a target to control hyperuricemia. In our study, DKB114 inhibited the in vitro XOD and hepatic XOD activity in rats with PO-induced hyperuricemia, which supports the findings of previous studies [18,19,20,21]. Furthermore, DKB114 decreased liver uric levels in hyperuricemic models, indicating that inhibition of XOD activity by DKB114 might be attributable to the reduction in uric acid production. Thus, it seems likely that the beneficial antihyperuricemic effects of DKB114 may be attributable, at least in part, to its inhibitory effects on XOD activity and reduction of hepatic uric acid production.

Genome-wide association studies have demonstrated that approximately 90% of hyperuricemia patients show underexcretion of uric acid [25]. Excretion of uric acid mainly occurs in the kidney and intestine. Handling of uric acid in the kidney is achieved through a complex interplay between the reabsorption and secretion of uric acid. Multiple transporters are involved in uric acid transport in the kidney. Among the transporters, URAT1 and GLUT9 are localized on apical and basolateral membranes of renal proximal tubule cells, respectively, and they mediate uric acid reabsorption through mediates the reabsorption of uric acid from the proximal tubule transport of uric acid from the kidney lumen to blood [26,27]. Uricosuric agents, Ben and probenecid, effectively decrease serum uric acid levels through uric acid reabsorption [28]. OAT1 and OAT3, localized in the basolateral membrane of proximal tubules, are responsible for uric acid transport from the blood to epithelial cells [25]. Since more than 90% of the excreted uric acid is reabsorbed and about 10% is excreted in the urine, it is accepted that regulation of uric acid reabsorption plays an important role in uric acid excretion [29,30]. Therefore, URAT1 and GLUT9 are considered attractive therapeutic targets for hyperuricemia. As mentioned above, DKB114 exhibits potential as a potent uricosuric agent. In the present study, we examined the effects of DKB114 on these renal transporters in vitro and in vivo. Interestingly, in hURAT1-expressing oocytes and HEK293 cells, DKB114 inhibited uric acid uptake into the cells without causing cytotoxicity. In addition, DKB114 decreased the URAT1 and GLUT9 protein expression levels in the kidney in rats with PO-induced hyperuricemia, although OAT1 and OAT3 protein expression levels did not change. These results suggest that reduction of cellular uric acid uptake by URAT1 transporters and decrease in the expression of renal transporters can inhibit uric acid reabsorption, which may contribute to the promotion of uric acid excretion and thus alleviate hyperuricemia.

Drugs to treat hyperuricemia and gout are currently available, but treatment failure and adverse effects has been reported [31]. Uricosuric drugs such as probenecid and Ben also increased the risk for kidney damage [32]. In particular, hepatotoxicity has been a big concern associated with Ben, although the extract mechanisms underlying its hepatotoxicity remain unclear. Recently, mitochondrial toxicity and reactive metabolite formation were proposed as potential mechanisms [33,34]. In our study, DKB114 did not decrease MP or cell viability of HepG2 cells, indicating that DKB114 probably did not have the potential to cause mitochondrial toxicity and has mild side effects.

There are limitations in our study; (1) DKB114 is crude extract of two different plant sources that might contain numerous components and compounds. (2) Effects of DKB114 is direct or indirect unknown. In our previous study, HPLC showed that DKB114 contains six components: Chlorogenic acid and 3,4-dicaffeoylquinic acid from CF and coumarin, cinnamaldehyde, trans-cinnamaldehyde, and *o*-methoxycinnamaldehyde from CB; these components exerted XOD inhibitory activity in vitro [21]. This suggests that these components may be partially responsible for the antihyperuricemic effects of DKB114. However, we did not examine whether these components enhance the excretion of uric acid through regulation of various transporters associated with uric acid excretion. Therefore, the mechanism underlying the promotion of uric acid excretion and analysis of bioactive compounds needs to be investigated further in future. 

## 5. Conclusions

The present study demonstrated that DKB114 reduced serum uric acid levels in normal rats and rats with PO-induced hyperuricemia and promoted the excretion of uric acid in urine, indicating that DKB114 has an antihyperuricemic effect and may be a potent uricosuric agent. In addition, DKB114 inhibited XOD activity and hepatic uric acid production in vitro and in vivo, as well as cellular uptake of uric acid in vitro. Furthermore, it decreased the protein expression levels of renal transporters such as URAT1 and GLUT9 in the kidney. Thus, the antihyperuricemic effects of DKB114 may be due, at least in part, to its inhibitory effects on XOD and reabsorption of uric acid. On the basis of these results, we propose that DKB114 exerts beneficial effects on hyperuricemia and may be useful in the treatment of hyperuricemia.
